# Supplementary material for: Sustained-input switches for transcription factors and microRNAs are central building blocks of eukaryotic gene circuits
Source: Genome Biol. 2013 Aug 23;14(8):R85. doi: 10.1186/gb-2013-14-8-r85 (PMC4054853; doi:10.1186/gb-2013-14-8-r85)
Supplement: Additional file 5 — HTML Browsable Motif Output. Zipped folder containing all WaRSwap and FANMOD motif output, viewable in a web browser. [file gb-2013-14-8-r85-S5.ZIP › HTML_browsable_motif_output/FANMOD_ath_tair9/sigs_fanmodm-2000.pvals.heatmaps.html/motif_id_46_000101110_tftype_ath_upstream_-3000_0.html]

```
BG_MODEL = FANMOD
MOTIF_ID = 46_000101110
TF_TYPE = ath
UPSTREAM = -3000_0


PVals
FN_0.2	FN_0.4	FN_0.6	FN_0.8
dg_60.genes	0.79	0.003	0.001	0.771
dg_70.genes	0.789	0.004	0	0.777
dg_80.genes	0.798	0.005	0	0.781

ZScores
FN_0.2	FN_0.4	FN_0.6	FN_0.8
dg_60.genes	-0.827	2.556	3.523	-1.231
dg_70.genes	-0.81	2.527	3.474	-1.27
dg_80.genes	-0.837	2.568	3.559	-1.259

StDevs
FN_0.2	FN_0.4	FN_0.6	FN_0.8
dg_60.genes	32.275	28.239	14.831	2.776
dg_70.genes	32.892	28.131	15.097	2.822
dg_80.genes	33.45	28.294	14.593	2.864
```
